# The College of Spirits in Paris

**Published:** 1858-09

**Authors:** 


THE COLLEGE OF SPIRITS IN PARIS.—We learn, from the *New York Tribune*, that Mr. D. D. Hume, the celebrated Spiritualist, is about marrying the sister-in-law of a Russian noble, and intends establishing a spiritual college in Paris. The particulars of the humbug we extract from the *Tribune*.

"Hume will return to Paris with his wife and the dowry, and they say that after the examples of Mesmer and Cagliostro, he is going to found at Paris a grand establishment of supernatural communications, a sort of Spiritualistic Exchange.

"To this establishment there will be joined a school of *Fluidism*, where the rich will be initiated in that great mystery—requiring a sacrifice proportioned to their wealth. This school will be divided into three classes. The first will be a sort of gymnasium, purely mechanical, where will be shown the method of disengaging the fluid by exercises at once physical and intellectual. Everybody possesses the spiritual fluid, and if some appear to be without it, it is because they do not know how to produce its disengagement. Hume said as much to us not long ago; and to make himself understood, he added the following explanation:—

"'Here is a cake of resin. This cake contains a great quantity of electricity. But this electricity does not manifest itself; it produces no phenomena; it sleeps. To awaken it I take this catskin and strike the cake of resin, and the electricity manifests its presence in a lively manner.'

"The first class of the school of fluids will be that in which they will operate on the natures possessing latent fluid, as they operate upon the resin with the catskin; accordingly, we call this the catskin class.

"In the second class, the fluid being developed, awakened, and active, they will show how to direct it by faith and by will. It is not sufficient to have the fluid, it must also be known how to use it.

"The mode of using it is, then, what they will teach in the second class. In leaving this class, the adepts will know how to turn tables, to summon spirits, to question them, to receive answers; and, in fact, to place themselves in communication with the other world. This is the class of Reception.

"But, when this is known, all is not done. This is only to be in communication with the spiritual world; it remains yet to learn how to profit by these communications. They must not be regarded as useless play; as a series of curious but unfruitful experiments. We must learn all that the spirits know more than we do; we must use them to elevate ourselves, to make us better, richer, and more powerful.

"That is what will be learned in the third class.

"Well-informed persons pretend that, before returning to Paris, Hume will pass through Holstein, where he will visit, in the cave whither he has retired, the celebrated Count of Saint Germain, from whom he expects to obtain (for the spirits have promised it him) twenty-seven of the fourteen thousand seven hundred secrets which the immortal Count carries in his bosom.

"These twenty-seven secrets — the most important of the ancient Egyptian Cabala, and which are to restore to us the mysteries of Isis and Anubis — these twenty-seven secrets, together with the four that Hume already knows, are to form a total of superhuman knowledge which will make the happy initiated equal in power, beauty, longevity, health, happiness, and knowledge, with the inhabitants of the planets of the third order. The earth, it is well known, is only a poor planet of the forty-fourth order.

"The third class will be called the class of Results.

"We are informed that while Mr. Hume will open his school of Fluidism for men, and will make the living talk with the dead, Madame Hume, on her side, will direct a similar school for females.

"The number of pupils can never exceed sixty on the part of the males, and sixty on the part of the females. Each class will be composed of thirty persons.

"It is pretended that a company, composed of some very wealthy Russians and some Frenchmen, is formed for the establishment of these institutions, and that they are now negotiating for the purchase of the lands of the Hotel d'Osmont, in Paris.

"When these two schools are finally opened, Paris will be really the capital of the world. The plans are already in preparation. Two temples are spoken of, of the Egyptian order, connected by a gallery, in the centre of which, beneath a circular pavilion, surmounted by a cupola, will be placed a large circular table, around which eighty-two persons of both sexes, in alternate order, can be seated. These eighty-two persons will be Mr. and Madame Hume, forty male pupils and forty female pupils. The scholars of the first class cannot assist in turning the sacred table.

"The table being set in motion, the spirits evoked, and the mysteries prepared, the twenty men and the twenty women of the second class will retire, and it is only for the initiated of the third class that the miracles will take place, and the eyes of the mind be opened."

